# Investigating *CENPW* as a Novel Biomarker Correlated With the Development and Poor Prognosis of Breast Carcinoma

**DOI:** 10.3389/fgene.2022.900111

**Published:** 2022-06-17

**Authors:** Luyang Wang, Hairui Wang, Chen Yang, Yunyi Wu, Guojie Lei, Yanhua Yu, Yan Gao, Jing Du, Xiangmin Tong, Feifei Zhou, Yanchun Li, Ying Wang

**Affiliations:** ^1^ Laboratory Medicine Center, Department of Clinical Laboratory, Zhejiang Provincial People’s Hospital (Affiliated People’s Hospital, Hangzhou Medical College), Hangzhou, China; ^2^ Department of Central Laboratory, Affiliated Hangzhou First People’s Hospital, Zhejiang University School of Medicine, Hangzhou, China; ^3^ School of Pharmacy, Hangzhou Medical College, Hangzhou, China; ^4^ School of Laboratory Medicine and Life Science, Wenzhou Medical University, Wenzhou, China; ^5^ Traditional Chinese Medicine Department, Zhejiang Provincial People’s Hospital (Affiliated People’s Hospital, Hangzhou Medical College), Hangzhou, China

**Keywords:** *CENPW*, poor prognosis, biomarker, breast cancer, therapeutic strategy

## Abstract

Breast invasive carcinoma (BRCA) is a carcinoma with a fairly high incidence, and the therapeutic schedules are generally surgery and chemotherapy. However, chemotherapeutic drugs tend to produce serious toxic side effects, which lead to the cessation of treatment. Therefore, it is imperative to develop treatment strategies that are more effective and have fewer side effects at the genetic level. Centromeric protein W (*CENPW*) is an oncogene that plays an important part in nucleosome assembly. To date, no studies have reported the prognostic significance of *CENPW* in breast carcinoma. In this study, we verified that *CENPW* expression is up-regulated in breast carcinoma and positively associated with the level of immune cell infiltration. The clinicopathological characteristics further suggest that *CENPW* expression is correlated with a worse prognosis of breast carcinoma. Interestingly, the *CENPW* mutation contributes to the poor prognosis. Next, we discovered that the genes interacting with *CENPW* are mainly concentrated in the cell cycle pathway, and *CENPW* is co-expressed with *CDCA7*, which is also highly expressed in breast carcinoma and leads to a worse prognosis. Our subsequent studies verified that knockdown of *CENPW* significantly inhibits the proliferation and migration of breast carcinoma cells and promotes their apoptosis rate. Notably, inhibition of CEMPW sensitizes breast cancer cells to chemotherapeutic drugs that have been found to induce cell cycle arrest. In summary, these results provide extensive data and experimental evidence that *CENPW* can serve as a novel predictor of breast cancer and may act as a prospective therapeutic target.

## Introduction

The International Agency for Research on Cancer (IARC) announced the latest global carcinoma burden data in 2020, showing that the new incidence of breast carcinoma in the world is up to 11.7%, which has overtaken lung carcinoma as the world’s largest most common cancer. Breast invasive carcinoma (BRCA) is the widest tumor type in women, with a poor prognosis and high recurrence rates (Lang et al., 2017). At present, paclitaxel combined with carboplatin has made great progress in the therapy of breast carcinoma, the long-term use of chemotherapeutic drugs produces obvious resistance and the therapeutic efficacy is still not ideal. (Fasching et al., 2021). In the general population, the pathogenesis of breast cancer is often directly related to alterations in gene expression (van den Broek et al., 2021). Therefore, finding the driver genes in tumorigenesis may open up new strategies for early breast cancer screening and the discovery of potential therapeutic targets, which will facilitate early detection and thereby reduce morbidity and mortality (Shen et al., 2021).

Centromeric protein W (*CENPW*), also known as cancer upregulated gene 2 (*CUG2*) protein, is overexpressed in multiple tumor tissues, including cervical, colon, liver, and lung cancers (Malilas et al., 2014). The *CENPW* is located at human chromosome 6q22.32 and is regulated by the upstream TSS region, which can transcribe a full-length mRNA of 600 bp (Lee et al., 2007). *CENPW* is a representative member of the Constitutive Centromere Associated Network (CCAN) family and is of crucial importance in the formation of centromeric nucleosomes (Klare et al., 2015). CENPW is encoded by *CENPW* and forms a DNA-binding heterodimer with CENPT (Prendergast et al., 2016), which is able to switch centromeric chromatin to a mitotic state (Prendergast et al., 2011). Previous studies have found that the regulation of *CENPW* can affect the migration of colon carcinoma cells and induce the epithelial-mesenchymal transition of lung carcinoma cells via *TGF-β* signaling (Malilas et al., 2013; Kaowinn et al., 2018). However, the role and value of *CENPW* in breast carcinoma remain unclear and require further study.

In the current study, several databases were used to evaluate the role of *CENPW* in breast carcinoma. We discovered that *CENPW*, which is highly expressed in breast carcinoma, is associated with a poor prognosis of breast carcinoma. We further found that *CENPW* mainly influences the cell cycle pathway, and the downregulation of *CENPW* expression could inhibit the migration and proliferation of breast cancer cells and promote apoptosis. Notably, we believe that *CENPW* could be a biomarker in the development process of breast carcinoma and propose that inhibition of *CENPW* may be a prospective strategy for inhibiting breast carcinoma development.

## Materials and Methods

### GEPIA Analysis Dataset

GEPIA is an interactive website server based on TCGA and GTEx databases, which provides us with customizable functional modules (http://gepia.cancer-pku.cn/). In this study, the *CENPW* expression was analyzed using GEPIA in different tumors and paracancerous tissues. The association between *CENPW* and the highly coexpressed gene (*CDCA7*) was evaluated according to the function of correlation analysis. In addition, the OS of *CDCA7* gene was evaluated by the survival plots module.

### Oncomine Database Analysis

The Oncomine Database is an openly available database for the analysis of *CENPW* expression levels in multiple tumor types. The difference of mRNA expression and co-expression of *CENPW* gene in breast carcinoma were studied by this database. The thresholds were set as two-fold change, top 10% gene rank and *p*-value = 1E-4.

### UALCAN Cancer Database Analysis

The UALCAN Cancer Database comprehensively contains TCGA dataset, CPTAC dataset, and CBTTC dataset. The *CENPW* expression in BRCA was verified and the relationship between clinicopathological features and *CENPW* expression was identified by the UALCAN Cancer Database.

### Bc-GenEx Miner Analysis

Bc-GenEx Miner is a full-featured online access database, which is specialized for breast cancer. According to this database, the *CENPW* expression in different clinical stages was mined, and the correlation between *CENPW* and the related genes was clustered.

### Kaplan–Meier Survival Analysis

To evaluate the prognostic significance of a particular gene, the patient samples were divided into two groups on the basis of the median expression. The prognostic significance of *CENPW* in breast carcinoma was evaluated according to overall survival (OS), distant metastasis-free survival (DMFS), and relapse-free survival (RFS) by Kaplan–Meier plotter. Log-rank *p*-value and HRs with 95% confidence intervals were set.

### cBioPortal Website Analysis

cBioPortal is a comprehensive and open website based on the TCGA database, which provides us with visual cancer gene mutation data (http://www.cbioportal.org/). The *CENPW* gene mutations were analyzed using seven breast cancer databases available on the cBioPortal website.

### UCSC Xena Website Analysis

The UCSC Xena website contains multiple genomic databases for researching correlations between genomic or phenotypic variables (https://xenabrowser.net/). The TCGA database related to breast cancer provided by UCSC Xena was used for data mining to construct a heat map of *CENPW* and *CDCA7*.

### GeneMANIA Website Analysis

The GeneMANIA site comprises a wealth of gene information, and can be used for functional analysis (http://www.genemania.org). Its prediction algorithm has high accuracy (Warde-Farley et al., 2010). Therefore, the gene clusters of mutual value with *CENPW* were predicted using this website.

### Metascape Online Analysis

Metascape Online (https://metascape.org/gp/index.html) can be employed for function enrichment of *CENPW*-related gene sets and PPI network analysis (Zhou et al., 2019). And we used MCODE1 to further analyze densely connected regions. *p*-value < 0.05 was set as a cutoff value (Lu et al., 2021).

### TIMER Website Analysis

The new version of TIMER 2.0 has seven main analysis systems, with gene and survival functions designed to assess the infiltration of multiple immune cells (https://cistrome.shinyapps.io/timer/) (Li et al., 2017). *CENPW* was selected to generate a scatter map through a gene module to observe the relevance between the expression of *CENPW* and the immune infiltration level in breast carcinoma.

### Cell Culture

Human breast carcinoma cell lines (MDA-MB-231, BT-549) were purchased from the Cell Bank of the Chinese Academy of Science (Shanghai, China). MDA-MB-231 cell was cultured in DMEM (Hyclone, Logan, UT, United States) with 10% FBS (Gibco, Grand Island, United States) at 37°C and 5% CO_2_. BT-549 cell was cultured in RPMI-1640 (Hyclone, Logan, UT, United States) with 10% FBS (Gibco, Grand Island, United States) at 37°C and 5% CO_2_.

### Cell Transfection

Two pairs of siRNAs targeting *CENPW*, and negative control siRNA-NC (si-NC) were synthesized (RiboBio, Guangzhou, China). MDA-MB-231 and BT-549 cells were plated with appropriate cell density, and further transfected corresponding siRNA (si-NC, si1-*CENPW* and si2-*CENPW*) by Lipofectamine™ 3000 (Invitrogen, Carlsbad, CA, United States).

### Western Blot Analysis

The transfected cells were lysed with RIPA buffer (Sigma-Aldrich, United States) to extract protein solution. The protein concentration was gauged with BCA kit (Boxbio Science and vernight with primary antibodies, then removed for (HRP)-conjugated secondary antibody incubation. The blots were visualized by ECL-Plus kit (Thermo Scientific, United States).

### Cell Proliferation Detection

Cell proliferation was detected by CCK-8 (Meilunbio, China). 5,000 transfected cells were inoculated into a 96-well plate (NEST Biotechnology). After culture for 0, 24, 48 and 72 h, 10 μL CCK-8 reagent was added into detecting well, and the optical density at 450 nm wavelength was detected.

### Clone Formation Assay

Transfected cells (500 cells/well) were seeded into 6-well plates. A second transfection was performed 7 days after the first one to improve the transfection efficiency. After 14 days, the colonies were fixed with paraformaldehyde and stained with crystal violet for 20 min. Whereafter, the field of vision was photographed by a light microscope (Olympus, Japan).

### Transwell Invasion Assay

The transfected cells were seeded into the upper chamber (Corning, United States), and the lower chamber was added with 500 μL 5% FBS complete medium. After 24 h of culture, the cells were fixed with paraformaldehyde solution and stained with crystal violet for 20 min. Finally, the invading cells were captured by a light microscope (Olympus, Japan).

### Wound Healing Assay

The transfected cells were seeded into 6-well plates and grew to around 90% confluence. The monolayer cells were then scratched with the tip of a 10 μL pipette. After 24 h of culture, the wound healing area was captured by a light microscope (Nikon, Japan).

### Flow Cytometry Analysis

The cell apoptosis was detected by flow cytometry using the Annexin V-FITC/PI apoptosis kit (MultiSciences, Hangzhou, China). The transfected cells (6×10^5^ cells/well) were cultured in 6-well plates for 48 h and collected in 1×Binding buffer, then Annexin V-FITC (5 μL), and PI (10 μL) were added for 10 min incubation in the dark and subjected to the flow cytometry (Beckman coulter, CA, United States).

### Statistical Analysis

All statistical computing was performed by GraphPad Prism 7.0. Results are expressed as mean ± SD. The discrepancies between two groups were accomplished by the student’s *t*-test. Statistical significance was established at *p < 0.05, **p < 0.01, compared to the corresponding control group.

## Results

### The Evaluation of *CENPW* Expression in Multiple Cancer Tissues

**FIGURE 1:**
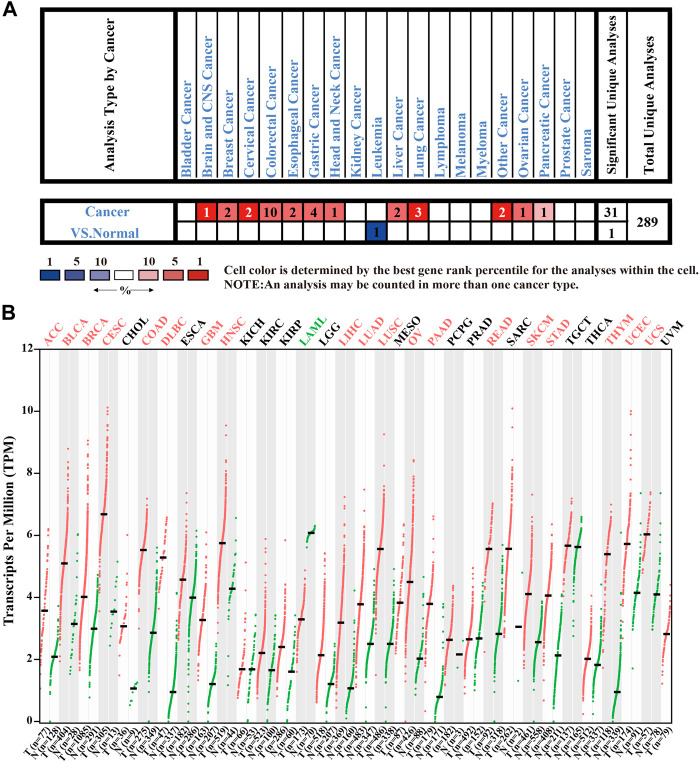
The expression levels of *CENPW* in different tissues. **(A)** The *CENPW* expression in multifarious tumor tissues and its corresponding normal tissues in the Oncomine database. **(B)** The *CENPW* expression in diverse types of carcinomas and normal tissues in GEPIA database.

To clarify the molecular biological characteristics of *CENPW* in different tumors, we first investigated *CENPW* expression in cancers and adjoining normal tissues from various databases. The data from the Oncomine database verified that the expression of *CENPW* was enhanced in numerous tumors including brain, breast, colorectal, head and neck, lung carcinomas compared with normal samples, while *CENPW* expression was lower in leukemia than in normal samples (Figure 1A). GEPIA database was further employed to evaluate the expression differences of *CENPW* in 33 kinds of tumor types (Figure 1B). We found that the *CENPW* was highly expressed in many tumors such as in BLCA, BRCA, COAD, HNSC, LUAD and STAD. On the contrary, the *CENPW* expression was lower than that of normal controls in acute myeloid leukemia. Collectively, these results from different databases together revealed that *CENPW* is excessively upregulated in most tumors and may act as a potential indicator of cancer progression.

### *CENPW* Acts as an Oncogene in the Carcinogenesis and Progression of Breast Carcinoma

**FIGURE 2:**
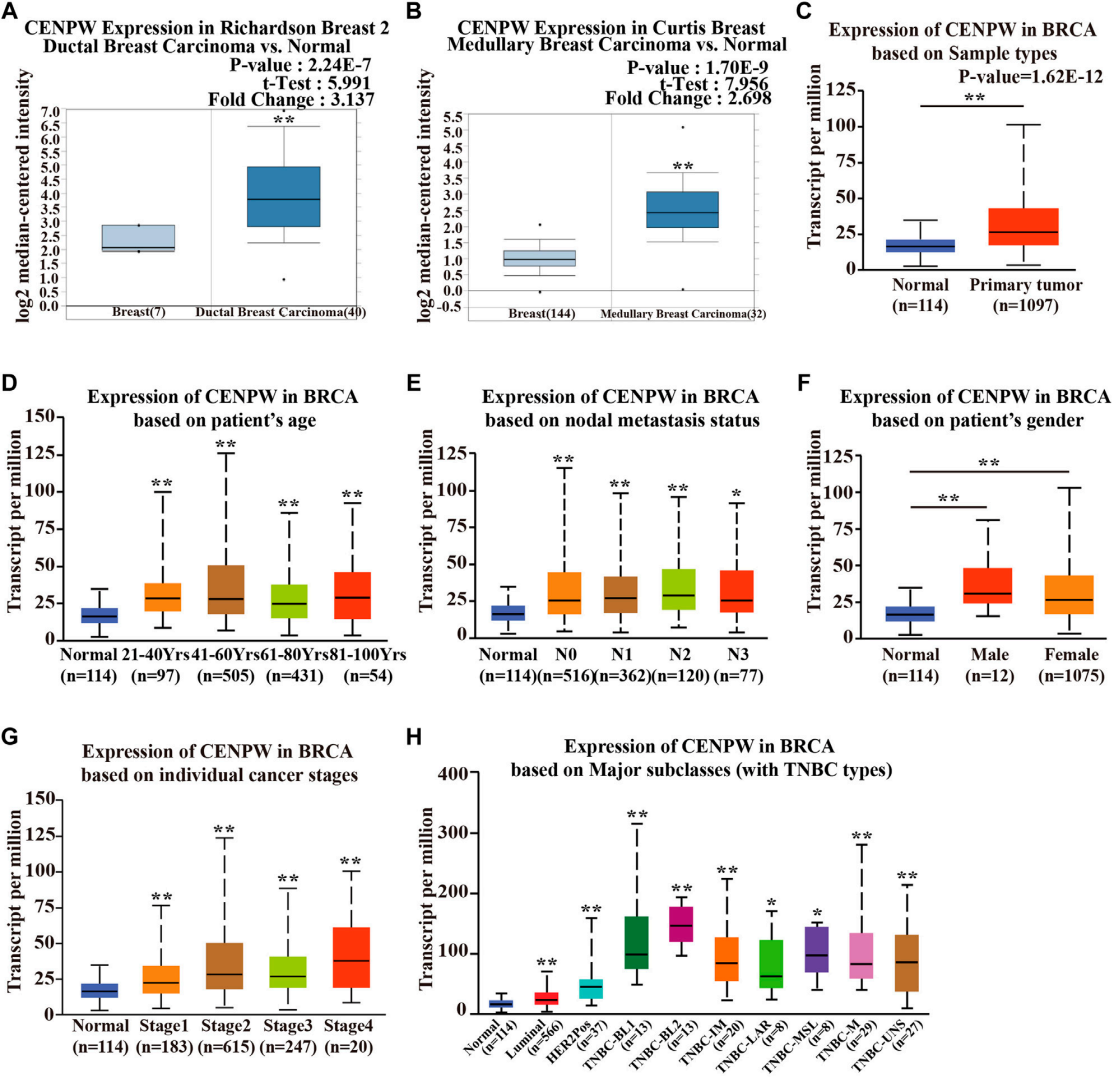
The *CENPW* expression in multiple subtypes of breast carcinoma. Expression levels of *CENPW* in **(A)** ductal breast carcinoma and **(B)** medullary breast carcinoma. **(C)** The *CENPW* expression in multiple subtypes of BRCA compared to normal samples derived UALCAN cancer database. Box plots of *CENPW* mRNA levels in the UALCAN cancer database based on **(D)** patient’s age, **(E)** gender, **(F)** nodal metastasis status, **(G)** individual cancer stages and **(H)** major subclasses (with TNBC types) of breast cancer patients were shown. *p < 0.05, **p < 0.01 compared with control group.

To better explore the potential value of *CENPW* in breast carcinoma patients, we further explored the expression and function of *CENPW* in breast carcinoma through several means. According to the Richardson Breast two dataset (Richardson et al., 2006), the *CENPW* expression in ductal breast cancer was distinctly higher than that in normal tissues (Figure 2A). In addition, the Curtis Breast dataset (Curtis et al., 2012) showed a 2.698-fold (*p* = 1.70E−09) increase in *CENPW* mRNA expression in medullary breast carcinoma (Figure 2B). Differences in mRNA expression of *CENPW* in breast carcinoma were also confirmed using the UALCAN database (Figure 2C). In addition, the UALCAN database was devoted to analyzing the relation between *CENPW* expression and different clinicopathological characteristics in BRCA patients. The data indicated that the *CENPW* expression was higher in female patients and was positively associated with patient age, nodal metastasis, and cancer stage (Figures 2D–G). Additionally, the relation between *CENPW* expression and triple-negative breast carcinoma classification was analyzed, and the expression level of *CENPW* was the highest in TNBC-BL2 (Figure 2H). The pairwise comparison *p-*values of the above results obtained by the UALCAN database are shown in Supplementary Table S1.

**FIGURE 3:**
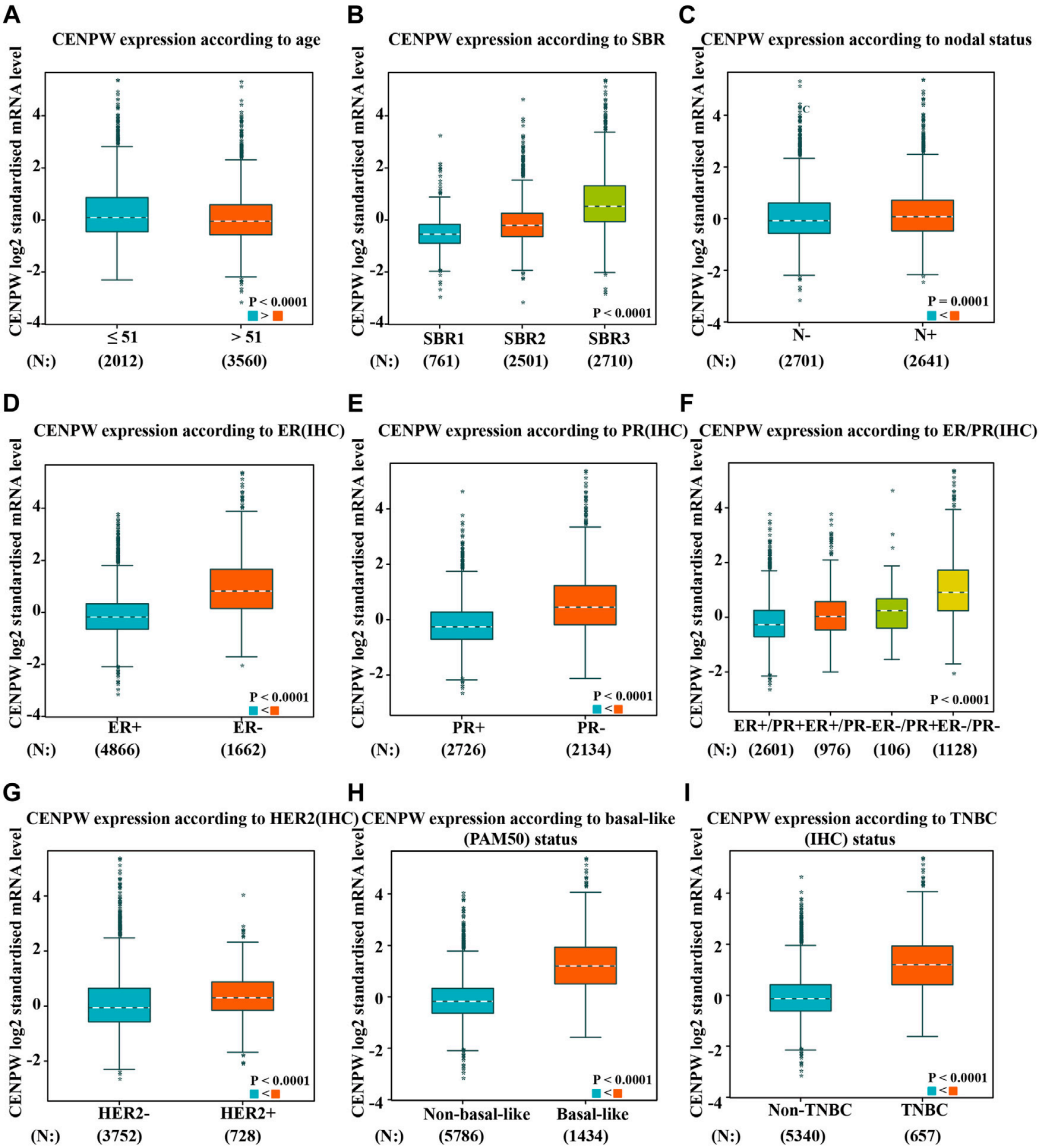
The correlation between *CENPW* expression and various clinicopathological parameters. Data are displayed for **(A)** age, **(B)** SBR, **(C)** nodal status, **(D)** ER, **(E)** PR, **(F)** ER/PR, **(G)** HER-2, **(H)** basal-like status and **(I)** triple-negative status.

We also evaluated the expression of *CENPW* with different clinicopathological features in breast carcinoma patients by the bc-GenExMiner online tool. The *CENPW* expression was higher in the less than 51-year-old group, and higher *CENPW* expression was correlated with a more advanced Scarff–Bloom–Richardson (SBR) grade (Figures 3A,B). *CENPW* levels in breast carcinoma patients with positive node status (N) were higher than those in patients with negative node status (Figure 3C). Progesterone receptor (PR) and estrogen receptor (ER) status were negatively correlated with *CENPW* expression (Figures 3D–F). In contrast, human epidermal growth factor receptor-2 (HER-2) status was positively correlated with *CENPW* expression (Figure 3G). In addition, *CENPW* increased in base-like subtypes compared to non-base-like subtypes (Figure 3H). Simultaneously, *CENPW* was strongly elevated in triple-negative breast cancer (TNBC) patients (Figure 3I). Detailed data on the clinicopathological features are presented in Supplementary Table S2.

### The High Level of *CENPW* is Correlated With Poor Prognosis in Breast Carcinoma Patients

**FIGURE 4:**
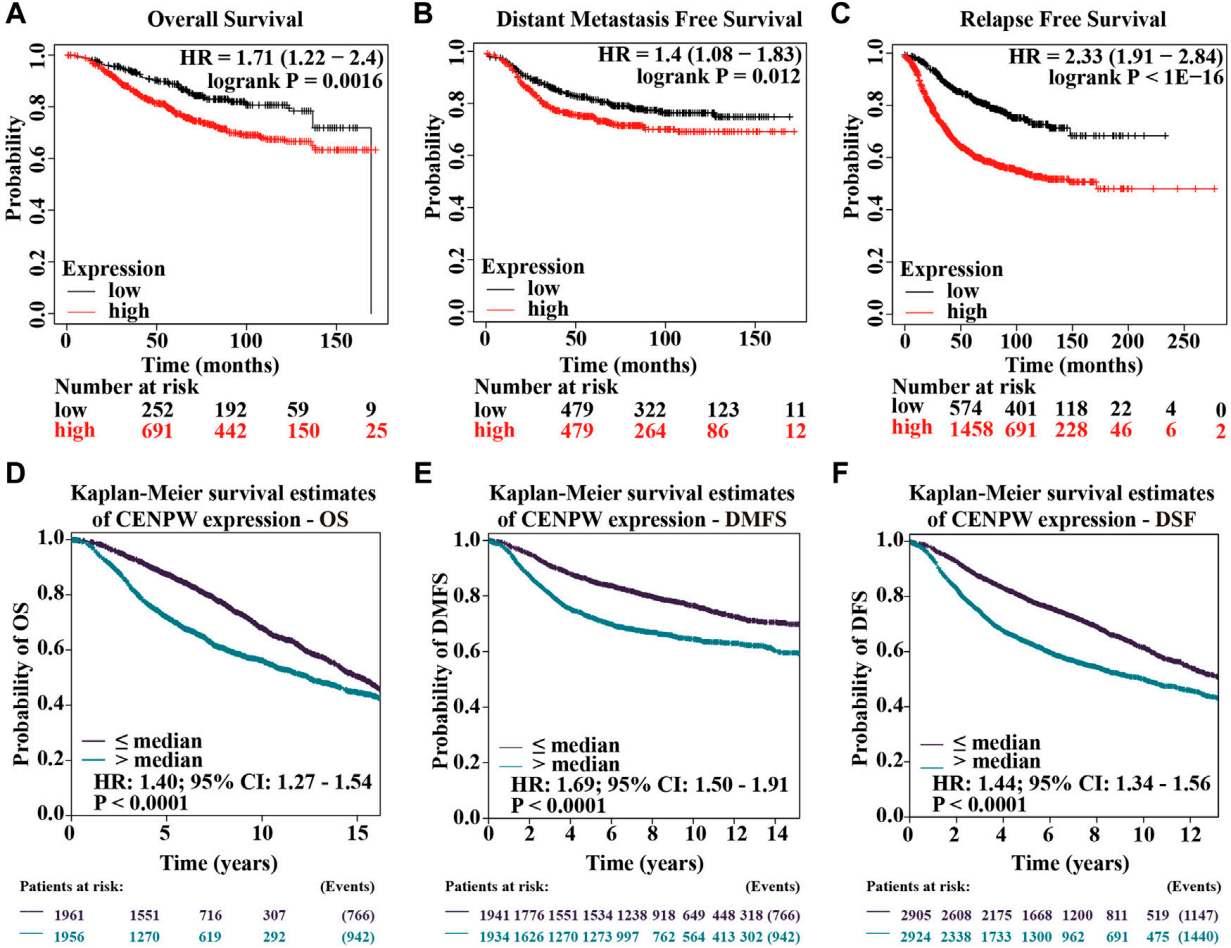
Survival data assessing the prognostic value of *CENPW*. Kaplan-Meier survival analysis of **(A)** OS, **(B)** DMFS and **(C)** RFS in breast cancer. And survival analysis of **(D)** OS, **(E)** DMFS and **(F)** DFS analyzed by bc-GenExMiner database in patients with breast cancer.

We further explored the relationship between *CENPW* expression and breast carcinoma prognosis. Results of the Kaplan-Meier plotter showed that higher expressions of *CENPW* were linked to poorer OS (Figure 4A). Similarly, DMFS and RFS were worse in breast cancer patients with increased *CENPW* expression (Figures 4B,C). To reconfirm the function of *CENPW* in breast carcinoma prognosis, we illustrated the effect of *CENPW* on OS, DMFS, and DFS by bc-GenExMiner database. Breast cancer patients with upregulated *CENPW* presented with worse OS, DMFS, and DFS (Figures 4D–F), which were similar to the results of the Kaplan-Meier plotter. In summary, these data showed that *CENPW* expression is associated with poor prognosis in breast cancer.

### The Analysis of Gene Mutation and Interaction of *CENPW* in Breast Carcinoma Patients

**FIGURE 5:**
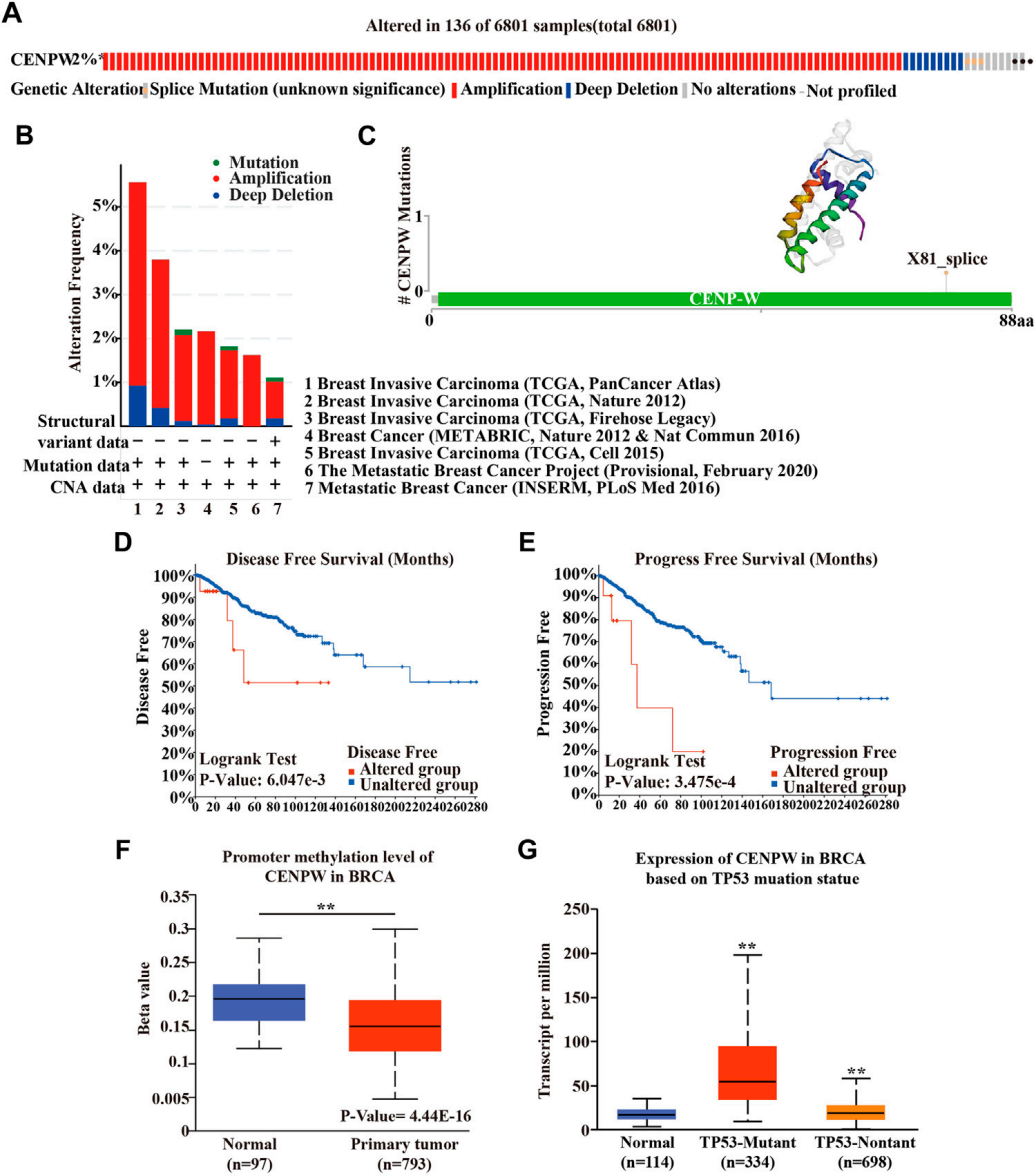
Gene mutation analyses of *CENPW*. **(A)** The mutation rate of *CENPW* in 6,801 samples. **(B)** Summary of alterations in various expressed *CENPW* in the cBioPortal database. **(C)** The mutation site and 3D structure of *CENPW*. Survival analysis of **(D)** DFS and **(E)** PFS of the mutated *CENPW* gene. **(F)** The promoter methylation level of *CENPW* and **(G)** the mRNA expression of *CENPW* based on TP53 mutation statue in BRCA by UALCAN cancer database. *p < 0.05, **p < 0.01 compared with control group.

To further understand the influence of *CENPW* gene mutations on breast carcinoma patients, we used the cBioPortal database and detected mutations in seven breast cancer databases. It was found that in 6,801 breast cancer patients, 2% had mutations in the *CENPW* gene (Figure 5A), and alterations with amplification were more common (Figure 5B). Furthermore, we predicted the 3D structure of *CENPW* and identified the mutation site using the cBioPortal online tool (Figure 5C). Next, we evaluated the effect of mutation on the prognosis of breast carcinoma patients and found that the high mutation group was obviously linked to shorter DFS and PFS in breast carcinoma patients (Figures 5D,E). In addition, the UALCAN database was used to assess the methylation levels of the *CENPW* promoters in BRCA (Figure 5F). The data indicated that the methylation level of the *CENPW* promoter in patients with BRCA was dramatically reduced, which may give rise to an increase in *CENPW* expression*.* We also revealed the relation between *CENPW* expression and TP53 mutations and found that *CENPW* was highly expressed in TP53 mutant groups (Figure 5G).

### The Co-expression of *CENPW* and *CDCA7* in Breast Carcinoma

**FIGURE 6:**
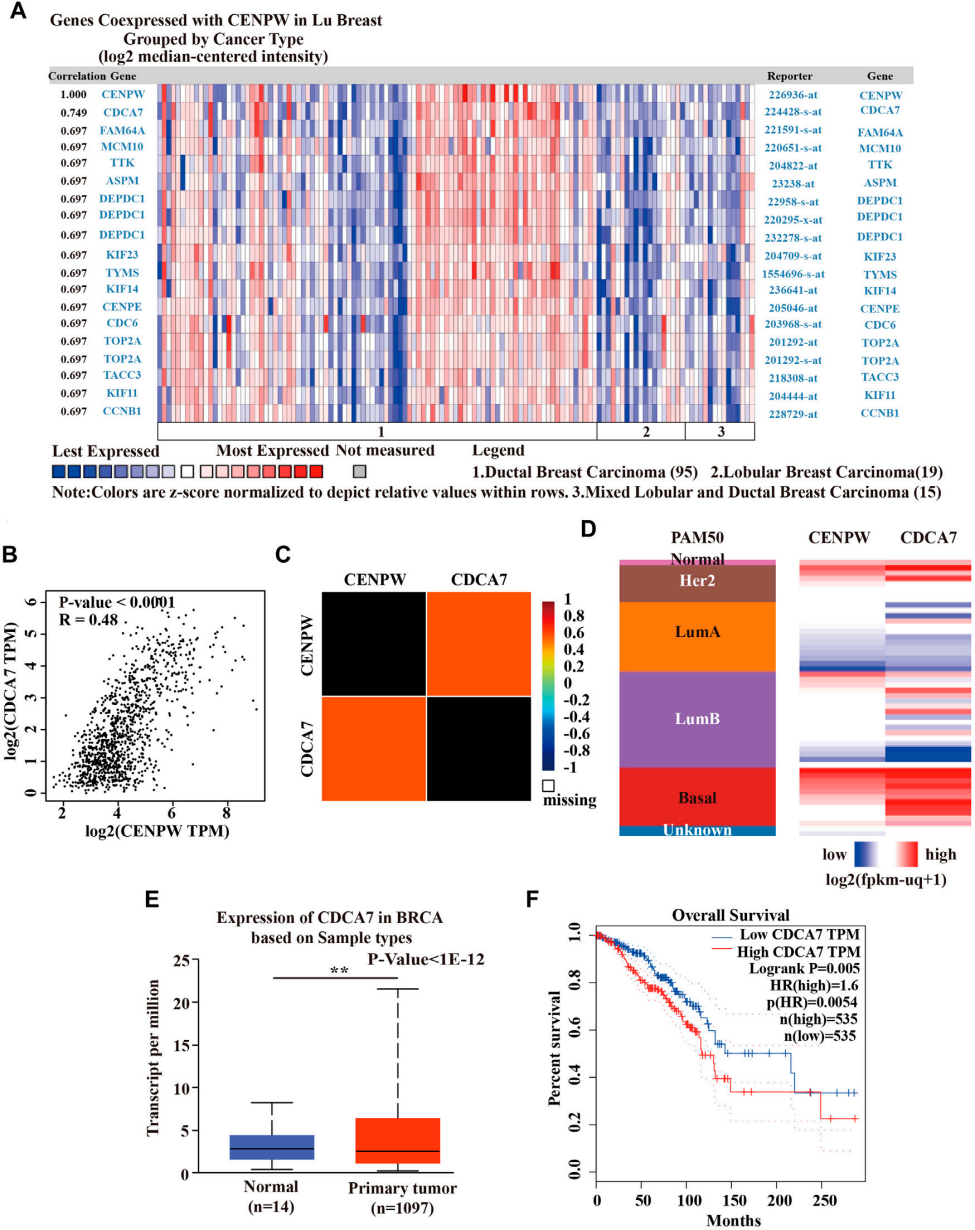
Coexpression analysis of *CENPW*. **(A)** Coexpression profile of *CENPW* derived from the Oncomine database. **(B)** The relation between *CENPW* and *CDCA7* expression in breast carcinoma in GEPIA website and **(C)** the bc-GenExMiner database. **(D)** Heat map of *CENPW* and *CDCA7* expression across PAM50 subtypes by UCSC Xena website. **(E)** The *CDCA7* expression of breast cancer in Ualcan database. **(F)** The OS status according to the *CDCA7* expression in GEPIA database. *p < 0.05, **p < 0.01 between the compared groups.

To further explore the mechanism of *CENPW* in BRCA, we employed the Oncomine database to construct the co-expression network of *CENPW* (Figure 6A). The co-expression profile showed that *CENPW* had the highest association coefficient with *CDCA7* among 129 breast carcinoma samples in the LU Breast dataset (Lu et al., 2008). We also confirmed the correlation between *CENPW* and *CDCA7* through the GEPIA website and bc-GenExMiner database and obtained results consistent with those of Oncomine (Figures 6B,C). Furthermore, *CENPW* and CDCA7 expression heat maps were derived using the UCSC Xena website. The *CENPW* expression was positively linked to the transcription level of *CDCA7* in a 50-gene qPCR assay (PAM50) for breast carcinoma subtypes (Figure 6D). These data indicated that *CENPW* might be associated with the *CDCA7* signaling pathway in breast cancer. Then the UALCAN database was performed to verify differences in *CDCA7* expression. The results revealed that *CDCA7* was obviously upregulated in BRCA (Figure 6E). According to the outcome of the GEPIA database survival analysis, *CDCA7* upregulation was correlated with poorer OS in breast carcinoma patients (Figure 6F), which was consistent with the effect of *CENPW* on breast cancer.

### The Enrichment and Interaction Analysis of *CENPW*-Related Genes

**FIGURE 7:**
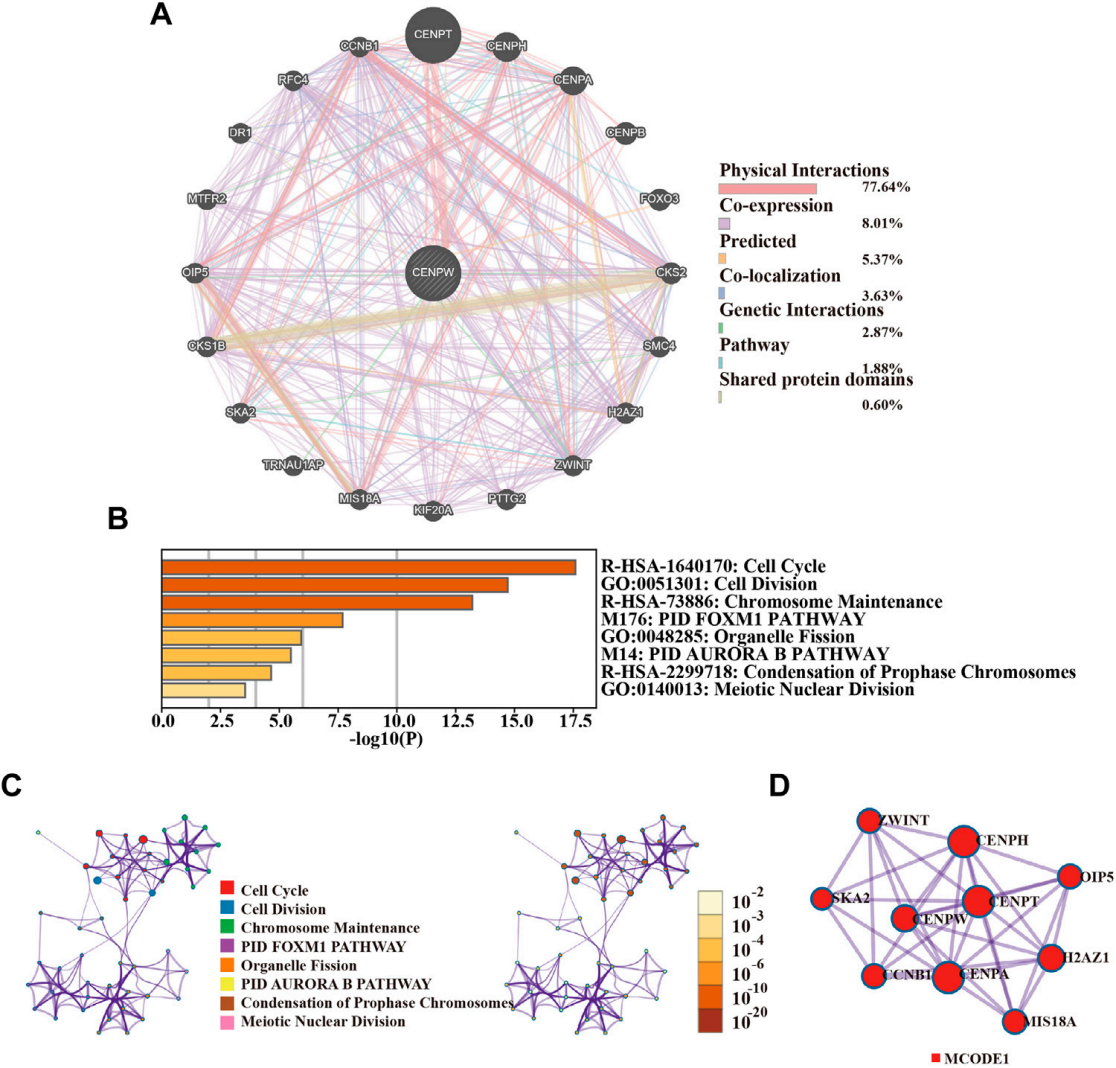
The enrichment and interaction of *CENPW* related genes. **(A)** PPI network of *CENPW* from the Metascape database. **(B)** Functional enrichment and signal path of the overlapping genes. **(C)** The GO and KEGG analysis suggesting the distribution and relation of the diverse functions according to Metascape Online database. **(D)** PPI network and MCODE showed associated genes interacting with *CENPW*.

Twenty genes (including *CENPT*, *CENPH*, *CENPA*, *FOXO3*, and *CKS2*) interacting with *CENPW* were excavated using the GeneMANIA website (Figure 7A). To explore the signaling pathway associated with *CENPW*, we used Metascape Online for functional analysis and discovered that the genes interacting with *CENPW* were primarily enriched in cell cycle, cell division, and chromosome maintenance (Figure 7B). These data inspired us to explore the relationship between *CENPW* and the cell cycle pathway. Next, the PPI network based on Metascape Online and the MCODE1 plug-in, further helped to identify these important modules of genes interacting with *CENPW* (Figures 7C,D). To further verify the co-expression of *CENPW* and *CDCA7*, we also conducted the above study on *CDCA7*. The functional analysis of twenty genes interacting with *CDCA7* showed that they were also mainly enriched in cell cycle signaling pathway (Supplementary Figure S1). This was consistent with the main biological effects of *CENPW* on breast cancer cells.

### The Correlation Between *CENPW* Expression and Immune Cell Infiltration

**FIGURE 8:**
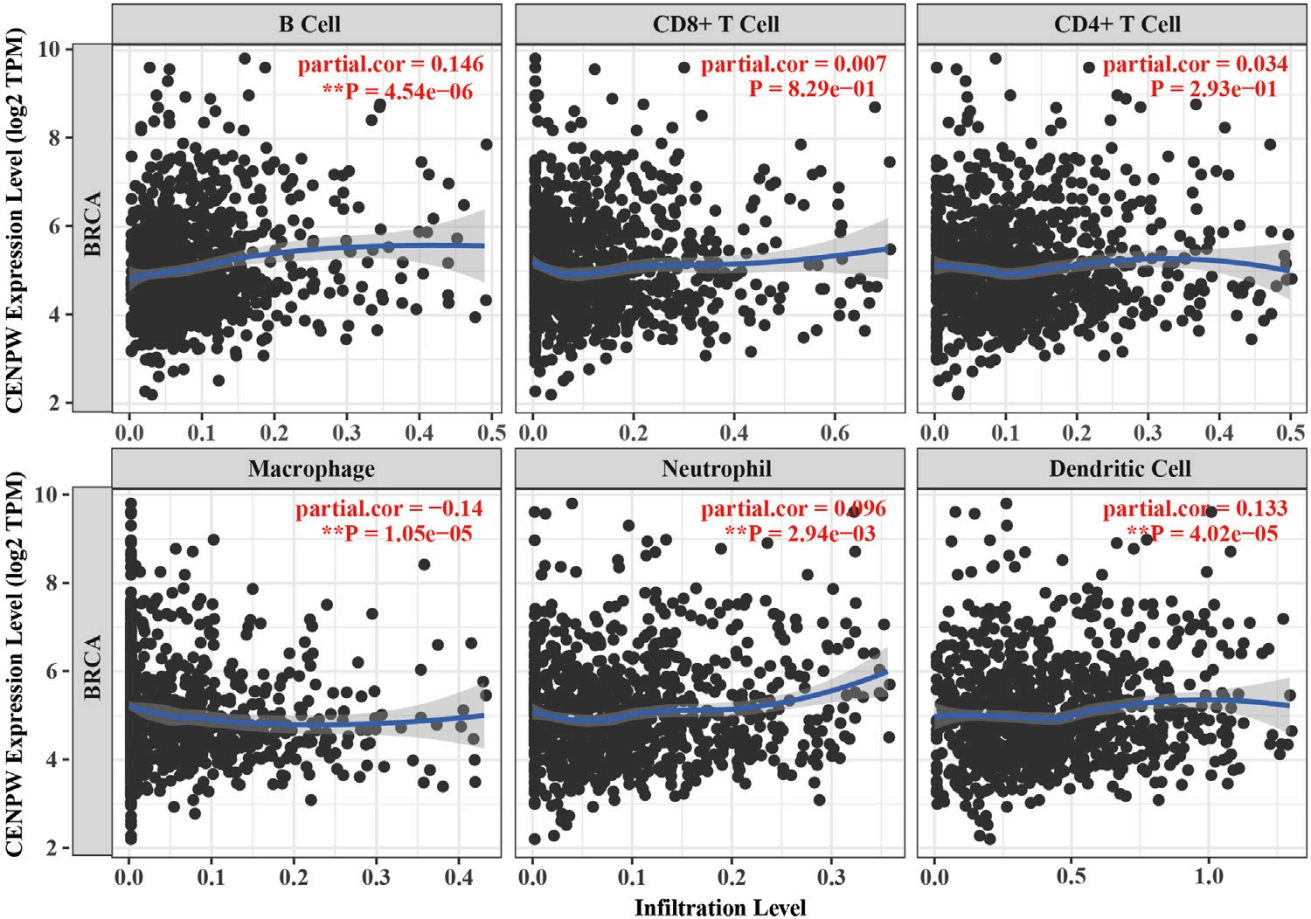
Immune infiltration data from the TIMER2.0 database. The relation between *CENPW* expression and the immune infiltration level in BRCA.

The relation between *CENPW* expression and the level of immune cell infiltration in BRCA was further investigated by TIMER2.0 database. Specifically, we investigated the association between *CENPW* expression and specific immune cell subsets. The data showed that *CENPW* expression was positively associated with most infiltrated immune cells in BRCA but negatively associated with the immune invasion levels of macrophages (Figure 8). There were noticeable discrepancies between *CENPW* expression and immune invasion levels of B cells, neutrophils, dendritic cells and macrophages. In addition, we analyzed the immune process of *CDCA7* in the breast cancer microenvironment, and the expression level of *CDCA7* was negatively correlated with macrophages, positively correlated with immune invasion of B cells, neutrophil, dendritic cell (Supplementary Figure S2), which is consistent with the results of *CENPW*.

### Knockdown of *CENPW* Inhibits the Proliferation of Breast Carcinoma Cells

**FIGURE 9:**
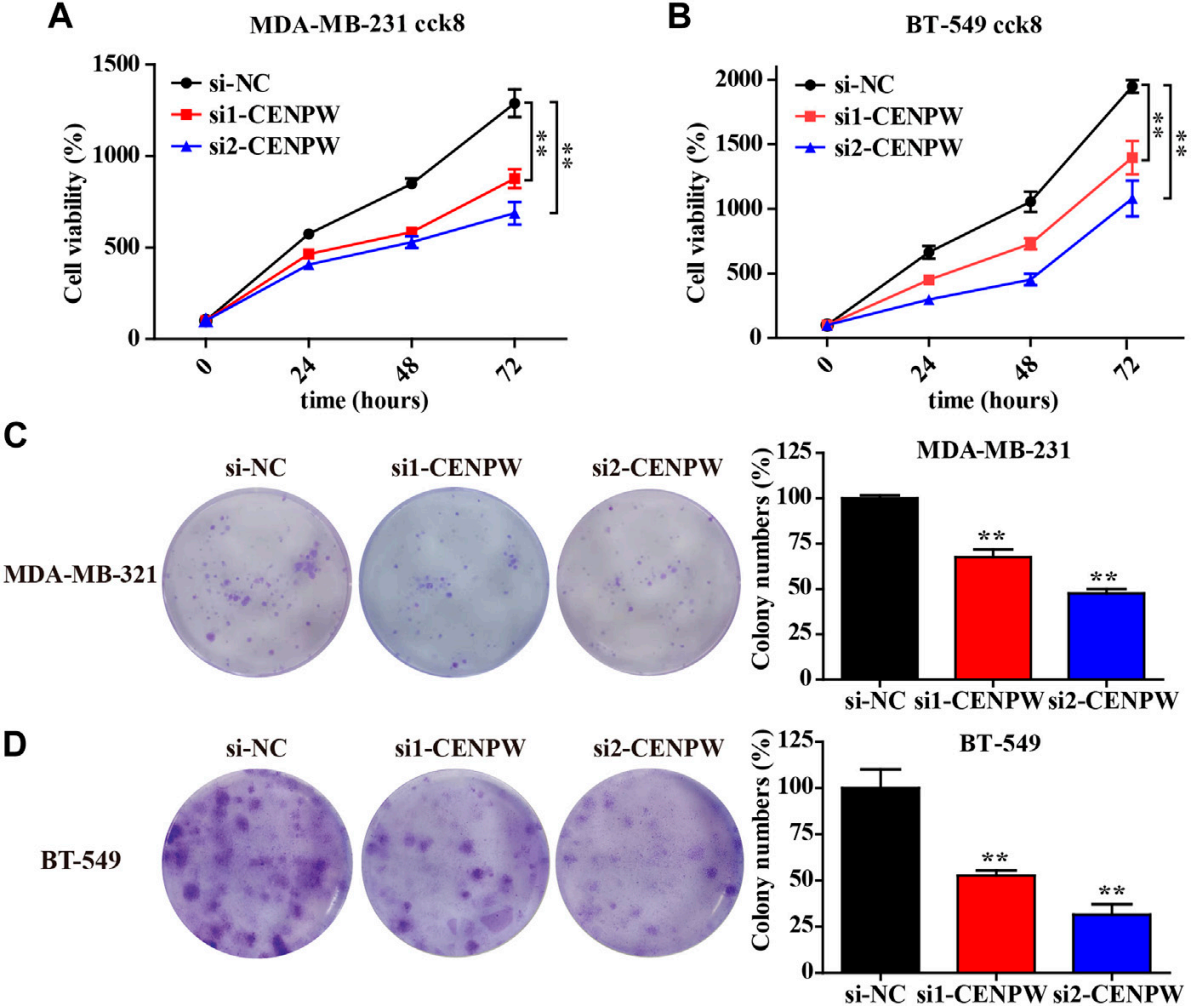
Knockdown of *CENPW* inhibits breast cancer cell proliferation. **(A,B)** Cell proliferation capability of control and *CENPW* knockdown cells. **(C,D)** The colony formation of breast cancer cells with or without *CENPW* knockdown. Quantitative histograms of colony formation are shown on the right. *p < 0.05, **p < 0.01 compared with si-NC group.

The above bioinformatics data proved that under-expression of *CENPW* was correlated with better survival in breast cancer patients. To explore the role of *CENPW* in breast cancer cells, we transfected MDA-MB-231 and BT-549 cells with two specific siRNA sequences to induce knockdown of *CENPW* expression. Growth curves suggested that *CENPW* knockdown suppressed the proliferation of these two breast carcinoma cell lines (*p < 0.05, Figures 9A,B).

In addition, we examined the effect of *CENPW* knockdown on long-term cell viability using a clonal formation survival assay in MDA-MB-231 and BT-549 cells (Figures 9C,D). And then after 14 days of culture, the clonal number of the two breast carcinoma cell treated with si1-*CENPW* and si2-*CENPW* decreased compared to the control cells.

### Knockdown of *CENPW* Inhibits Migration and Invasion of Breast Cancer Cells

**FIGURE 10:**
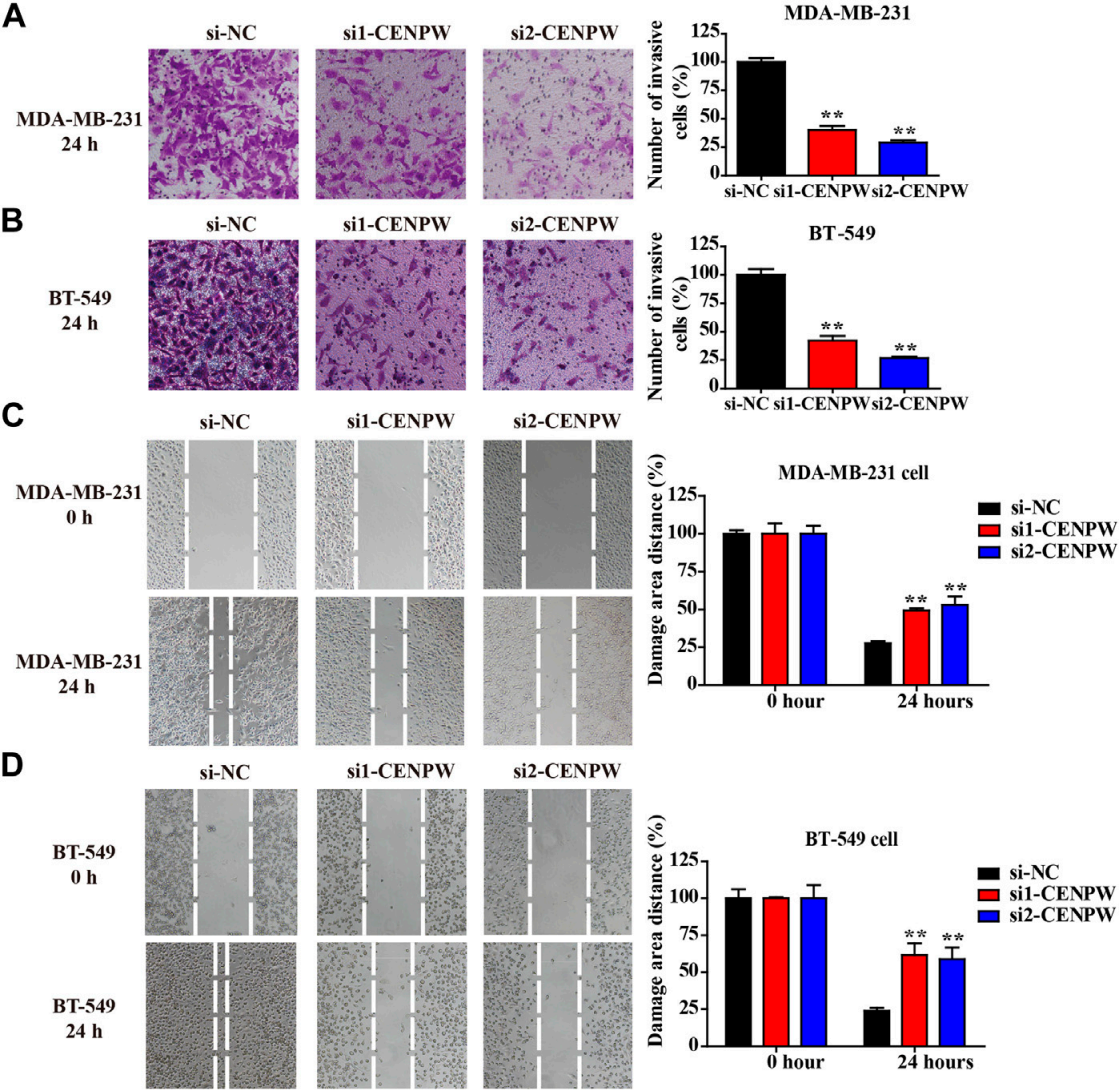
Knockdown of *CENPW* suppresses breast carcinoma cell invasion and migration. **(A,B)** The invasive ability was evaluated by transwell assay. **(C,D)** The migration capacity of control and *CENPW* knockdown cells was detected by wound healing assay. The statistical column diagrams are displayed on the right. *p < 0.05, **p < 0.01 compared with si-NC group.

The most concerning aspect of cancer is its ability to migrate and invade. Therefore, the invasion and migration of MDA-MB-231 and BT-549 cells were evaluated using adherent cell scratch migration and transwell invasion assays. Transwell analysis indicated that knockdown of *CENPW* reduced the number of invading cells (**p < 0.01, Figures 10A,B). In addition, as indicated by the scratch test in Figures 10C,D, the wound healing rates of MDA-MB-231 and BT-549 cells after *CENPW* silencing were significantly reduced (**p < 0.01). Moreover, these results together revealed that knockdown of *CENPW* can reduce the migration and invasion ability of breast carcinoma cells.

### Knockdown of *CENPW* Blocks the Cell Cycle Process and Increases the Probability of DNA Damage and Apoptosis in Breast Carcinoma Cells

**FIGURE 11:**
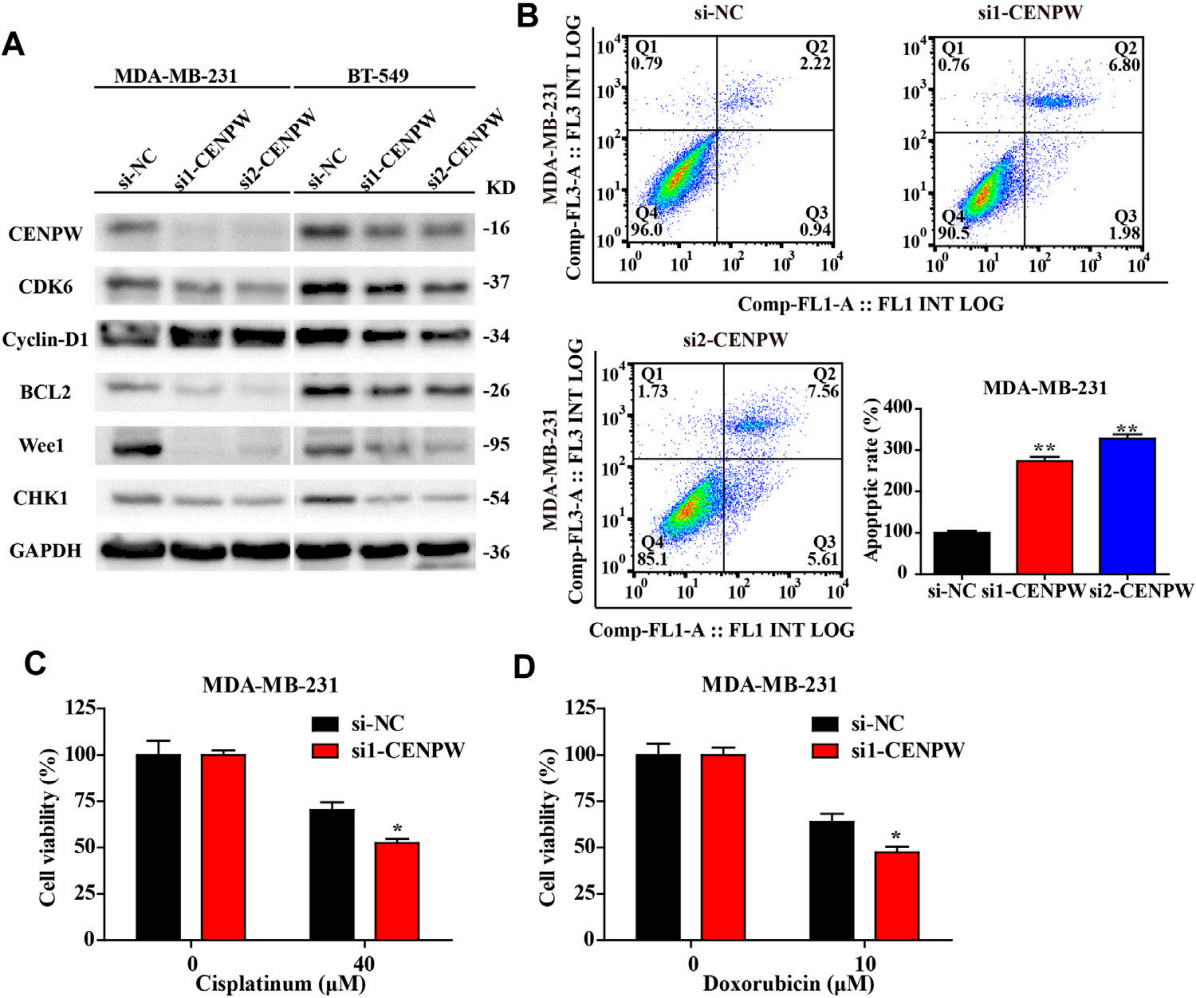
Knockdown of *CENPW* decreased the expression level of related pathway proteins and increased the apoptosis rate of breast carcinoma cells. **(A)** Western blot assay of control and *CENPW* knockdown cells. **(B)** The apoptosis rate was assessed by flow cytometry. **(C,D)** The cytotoxicity of cisplatin and doxorubicn in the *CENPW* inhibited breast carcinoma cells. *p < 0.05, **p < 0.01 compared with si-NC group.

To figure out the causes of these phenomena, we discovered the protein expression levels of the related pathways. The western blot results indicated that si1-*CENPW* and si2-*CENPW* could induce the degradation of CDK6 and Cyclin-D1, suggesting that inhibition of *CENPW* could block the cell cycle process, which was consistent with the data of GO and KEGG analysis. Furthermore, the significantly decreased protein expression levels of Wee1 and CHK1 indicate that knockdown of *CENPW* may cause DNA damage, which may be closely related to the participation of *CENPW* in the nucleosome assembly process. Meanwhile, we discovered that the expression of the apoptosis-related gene BCL2 decreased after *CENPW* knockdown (Figure 11A). Further apoptosis assay detected by flow cytometry reflected that knockdown of *CENPW* increased the proportion of apoptotic cells (Figure 11B). Notably, we found that breast cancer cells with reduced *CENPW* expression were more sensitive to chemotherapeutic drugs that have been found to induce cell cycle arrest. Improved tumor cell killing effect of cisplatin and doxorubicin was found in the *CENPW* inhibited breast carcinoma cells (Figures 11C,D).

## Discussion

At present, breast cancer has become the largest number of diagnoses and the highest incidence in women, and it has serious adverse effects on health (Joko-Fru et al., 2021). Therefore, it is crucial to investigate the pathogenesis, diagnosis, treatment, and prognosis of breast carcinoma (Liang et al., 2020). In the study, we explored and revealed that abnormal *CENPW* expression is closely linked to breast tumors through various databases and *in vitro* cell experiments. Ziliang Zhou, et al. (Zhou et al., 2020) reported that the *CENPW* expression level in liver carcinoma was obviously higher than normal liver tissues. Similarly, we discovered that *CENPW* expression was up-regulated in ductal breast carcinoma and medullary breast carcinoma using two different online databases. According to the clinicopathological features of breast carcinoma, compared with normal tissue, the HER-2 status, SBR grade, basal-like status, nodular status, and triple-negative status were positively associated with *CENPW* levels in breast carcinoma samples. In contrast, age, ER, and PR were negatively correlated with *CENPW* expression. These results indicated that *CENPW* expression was linked to poor prognosis of breast carcinoma, which was also confirmed by subsequent data from the bc-GenexMiner database. Therefore, based on the above series of data analyses, we believe that *CENPW* may be a promising indicator for screening and may be used as a new prognostic factor for breast carcinoma.

*CENPW* is a crucial member of the CCAN family, which plays a critical role in nucleosome assembly and is related to the deregulation of centromere proteins and carcinogenesis (Hori et al., 2008). Our findings showed that breast carcinoma patients with high *CENPW* expression had a worse prognosis than those with low *CENPW* expression. Notably, through gene mutation analysis in the cBioPortal database, we identified that mutations in *CENPW* were associated with poor prognosis. Interestingly, a highly correlated gene, cell division cycle-associated protein 7 (*CDCA7*) (Ye et al., 2018), was discovered by *CENPW* co-expression analysis. Cai et al. (Cai et al., 2021) reported that downregulation of *CDCA7* not only inhibits cell proliferation, but also intercepts the cell cycle in ovarian carcinoma. In the current study, functional enrichment analysis indicated that genes interacting with *CENPW* were mainly enriched in the cell cycle. These data indicated that *CENPW* and *CDCA7* may play a joint role in the cell cycle pathway; however, further direct evidence is required to confirm this. In addition, studies on *CDCA7* have found that its high expression can help to predict poor prognosis and tumor progression in colorectal carcinoma and kidney carcinoma (Li et al., 2020; Liu et al., 2021). Further investigation reveals that *CDCA7* expression is also up-regulated in breast carcinoma and leads to poor prognosis.

Notably, *CENPW* principally affects the cell cycle process of tumor cells by regulating nucleosome assembly (Sridhar et al., 2021). Therefore, we used the GeneMANIA website and Metascape online tools to analyze the gene enrichment and pathway enrichment of *CENPW*, and the data suggested that *CENPW* has a vital influence on the cell cycle pathway. In addition, Zhou et al. (Zhou et al., 2021) reported that knockdown of *CENPW* restrains HCC progression by inactivating *E2F* signaling. However, the significance of its downregulation in breast carcinoma prognosis has not yet been studied. To illustrate the specific molecular function of the *CENPW* gene in breast cancer, we constructed *CNEPW* gene silenced cells and found that *CENPW* suppression could inhibit the proliferation and migration of MDA-MB-231 and BT-549 cells, hindering the cell cycle signaling pathway, thereby causing apoptosis of breast tumor cells. Previous studies have confirmed that CENPW accelerates cell transformation and stemness through nuclear NPM1 protein and TGF-β signaling (Kaowinn et al., 2019a; Yawut et al., 2020). Moreover, Kaowinn et al. (Kaowinn et al., 2019b) found that CENPW elevated the expression of YAP1, resulting in the increase of the EMT in lung cancer cells. Interestingly, we found that breast cancer cells with *CENPW* inhibited were more sensitive to chemotherapeutic drugs that have been found to induce cell cycle arrest. These effects may be resulting from the inhibition of TGF-β signaling pathway, but required further exploration.

These cellular results were consistent with the above bioinformatics analysis and fully illustrated the fact that *CNEPW* may be a crucial oncogene and accelerant for the progression of breast carcinoma, which could help in the development of new breast carcinoma treatment strategies. However, there are still some limitations in our current study. A gene regulates multiple metabolic pathways, but its specific mechanism of action cannot be clearly explained by a single signaling pathway. Therefore, further experimental studies are required to better understand its functions. Further clinical trials are indispensable for elucidating the value of *CENPW* in the treatment of breast carcinoma.

## Conclusion

This study presents evidence that high *CENPW* expression in breast cancer generates poor prognosis and can therefore be used as a novel biomarker for breast carcinoma. In particular, mutations in the *CENPW* gene also led to a poor prognosis. Therefore, knockdown *CENPW* expression can suppress the proliferation and migration of breast cancer cells and induce apoptosis. Our results provide bioinformatics data and experimental evidence that *CENPW* can be used as a unique biomarker for breast carcinoma, and as a hopeful therapeutic option for the development of related inhibitors to treat breast cancer at the genetic level.

## Data Availability

The original contributions presented in the study are included in the article/Supplementary Material, further inquiries can be directed to the corresponding authors.
