# Supplementary material for: Investigating CENPW as a Novel Biomarker Correlated With the Development and Poor Prognosis of Breast Carcinoma
Source: Front Genet. 2022 Jun 17;13:900111. doi: 10.3389/fgene.2022.900111 (PMC9247308; doi:10.3389/fgene.2022.900111)
Supplement: Supplementary file 1 [file Table1.DOCX]

Table S1. *CENPW* expression in BRCA based on clinicopathological features by UALCAN database

| Clinicopathological Features | Comparison | Statistical Significance | Comparison | Statistical Significance |
| --- | --- | --- | --- | --- |
| Gender | Normal-vs-  Male | 7.16E-03 | Normal-vs-  Female | 1.62E-12 |
| Age | Normal-vs-  Age (21-40Yrs) | 1.94E-05 | Normal-vs-  Age (41-60Yrs) | <1E-12 |
|  | Normal-vs-  Age (61-80Yrs) | <1E-12 | Normal-vs-  Age (81-100Yrs) | 2.27E-05 |
| Nodal Metastasis Status | Normal-vs-  N0 | <1E-12 | Normal-vs-  N1 | 1.62E-12 |
|  | Normal-vs-  N2 | 3.11E-10 | Normal-vs-  N3 | 1.32E-02 |
| Individual Cancer Stages | Normal-vs-  Stage1 | 2.37 E-07 | Normal-vs-  Stage2 | 1.62 E-12 |
|  | Normal-vs-  Stage3 | 1.13E-09 | Normal-vs-  Stage4 | 6.79E-04 |
| Major Subclasses (with TNBC types) | Normal-vs-  Luminal | 1.62E-12 | Normal-vs-  HER2Pos | 2.44E-05 |
|  | Normal-vs-  TNBC-BL1 | 7.17E-04 | Normal-vs-  TNBC-BL2 | 2.62E-03 |
|  | Normal-vs-  TNBC-IM | 9.26E-07 | Normal-vs-  TNBC-LAR | 1.01E-02 |
|  | Normal-vs-  TNBC-MSL | 2.18E-02 | Normal-vs-  TNBC-M | 5.88E-05 |
|  | Normal-vs-  TNBC-UNS | 3.48E-06 |  |  |
