# Supplementary figures and images for: Investigating CENPW as a Novel Biomarker Correlated With the Development and Poor Prognosis of Breast Carcinoma
Source: Front Genet. 2022 Jun 17;13:900111. doi: 10.3389/fgene.2022.900111 (PMC9247308; doi:10.3389/fgene.2022.900111)

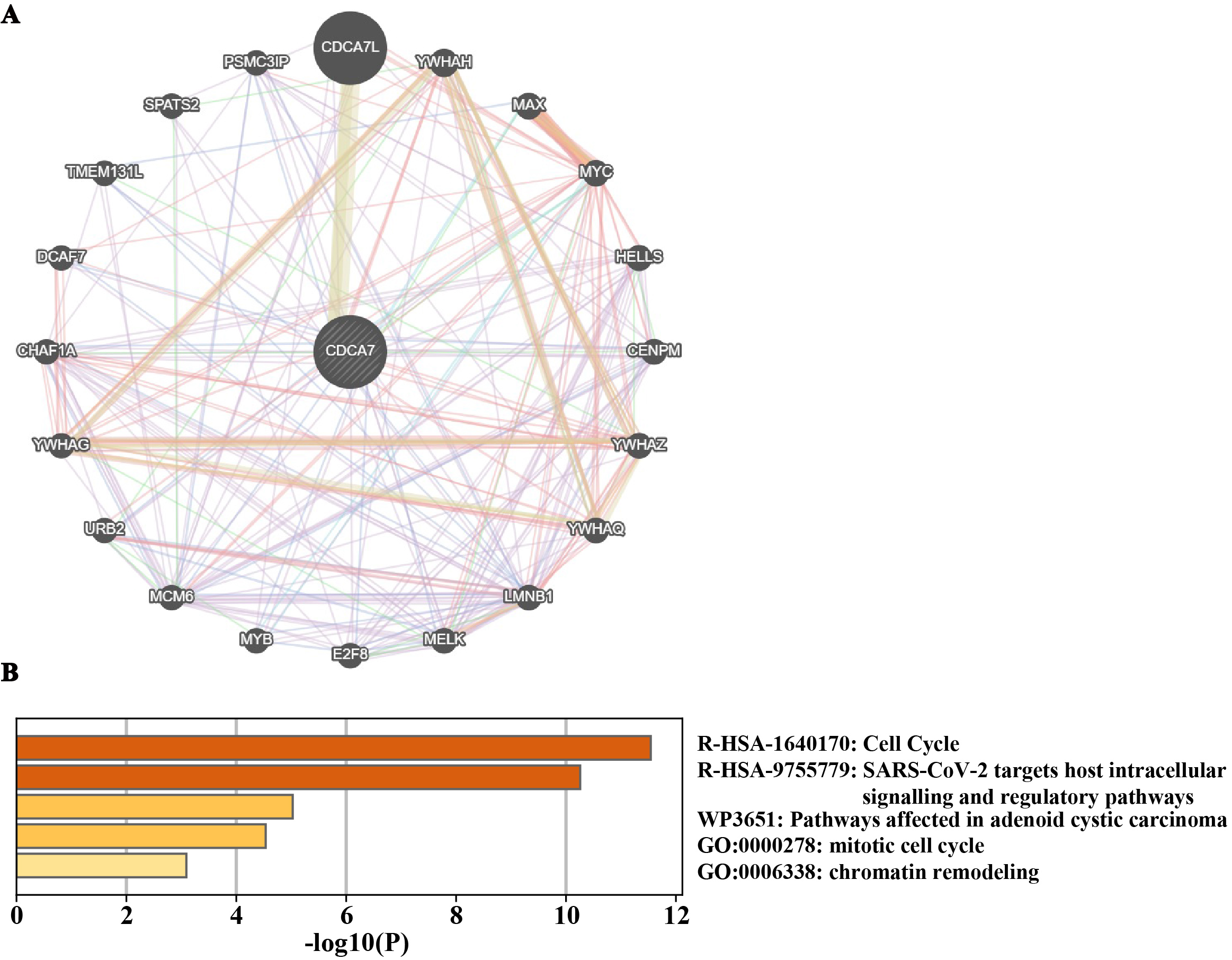

Supplement: Supplementary file 2 [file Image1.JPEG]

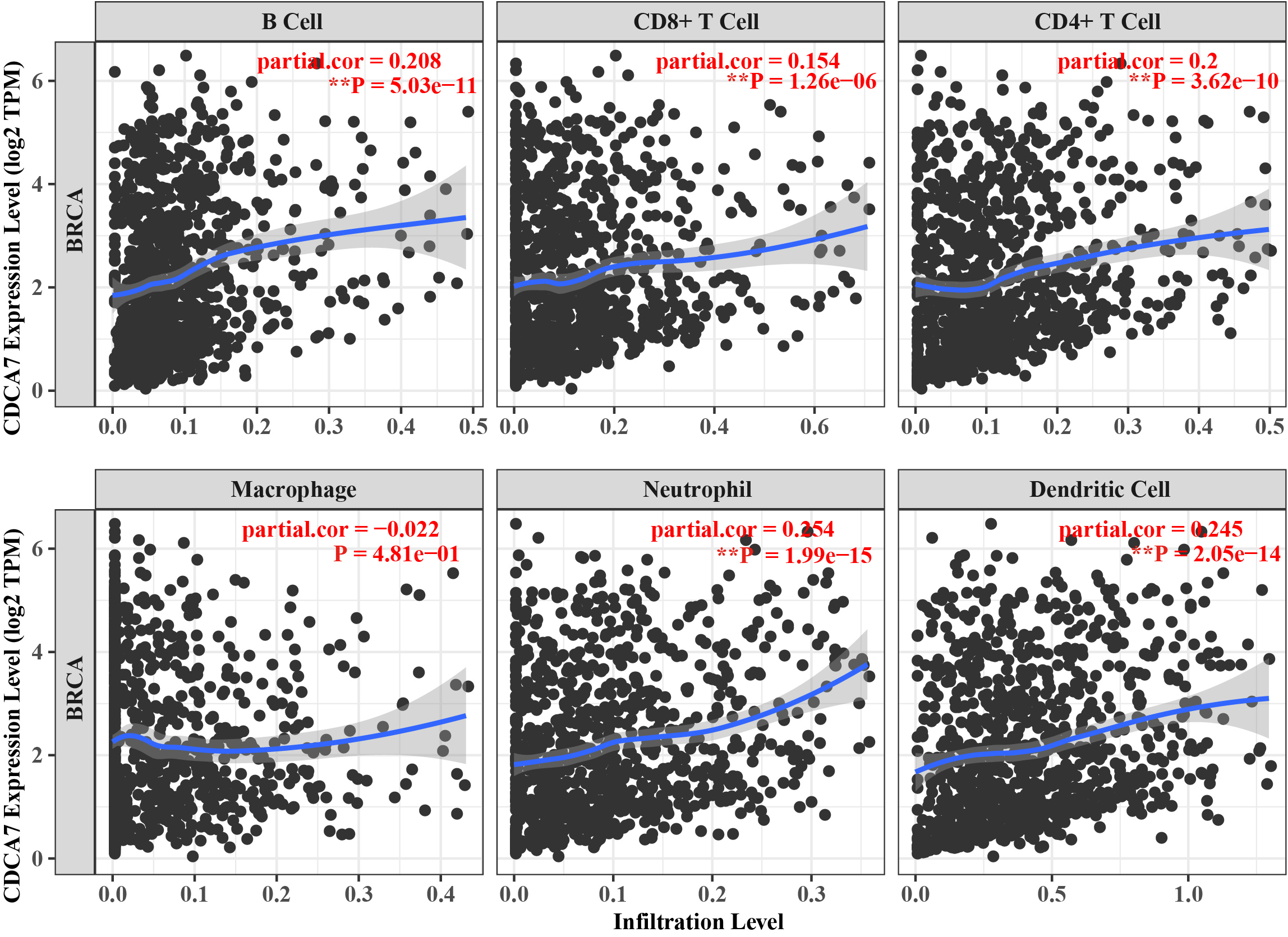

Supplement: Supplementary file 3 [file Image2.JPEG]
