# Supplementary material for: Investigating CENPW as a Novel Biomarker Correlated With the Development and Poor Prognosis of Breast Carcinoma
Source: Front Genet. 2022 Jun 17;13:900111. doi: 10.3389/fgene.2022.900111 (PMC9247308; doi:10.3389/fgene.2022.900111)
Supplement: Supplementary file 4 [file Table2.docx]

Table S2. Relationship between *CENPW* expression and clinicopathological features in breast cancer patients based on bc-GenExMiner database

| Variables | Patient number | mRNA | *P*-value |
| --- | --- | --- | --- |
| Age (years) |  |  | <0.0001 |
| ≤51 | 2012 | Increased |  |
| >51 | 3560 |  |  |
| SBR |  |  | <0.0001 |
| SBR1 | 761 |  |  |
| SBR2 | 2501 |  |  |
| SBR3 | 2710 | Increased |  |
| Nodal status |  |  | =0.0001 |
| Negative | 2701 |  |  |
| Positive | 2641 | Increased |  |
| ER |  |  | <0.0001 |
| Negative | 1662 | Increased |  |
| Positive | 4866 |  |  |
| PR |  |  | <0.0001 |
| Negative | 2134 | Increased |  |
| Positive | 2726 |  |  |
| HER-2 |  |  | <0.0001 |
| Negative | 3752 |  |  |
| Positive | 728 | Increased |  |
| Basal-like status |  |  | <0.0001 |
| Non-basal-like | 5786 |  |  |
| basal-like | 1434 | Increased |  |
| Triple-negative status |  |  | <0.0001 |
| Non-triple-negative | 5340 |  |  |
| Triple-negative | 657 | Increased |  |
